# Supplementary figures and images for: The mitochondrial genomes of two walnut pests, Gastrolina depressa depressa and G. depressa thoracica (Coleoptera: Chrysomelidae), and phylogenetic analyses
Source: PeerJ. 2018 Jun 5;6:e4919. doi: 10.7717/peerj.4919 (PMC5993032; doi:10.7717/peerj.4919)

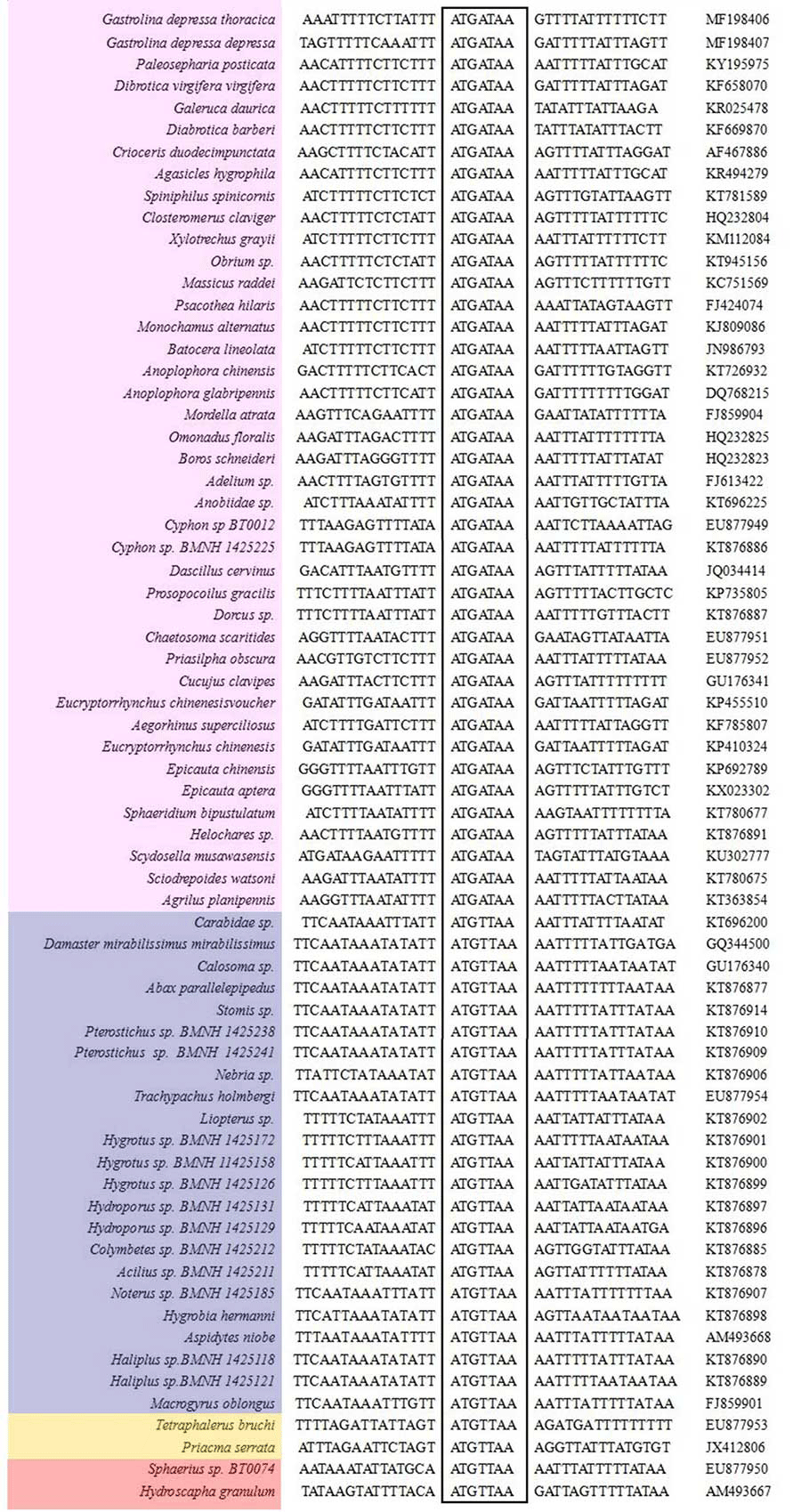

Supplement: Figure S1 — The boxed nucleotides indicate the 7 bp conserved overlaps (ATGTTAA/ATGATAA). Purple, blue, yellow and red represented species in the Polyphaga, Adephaga, Archostemata and Myxophaga, respectively. [file peerj-06-4919-s001.png]

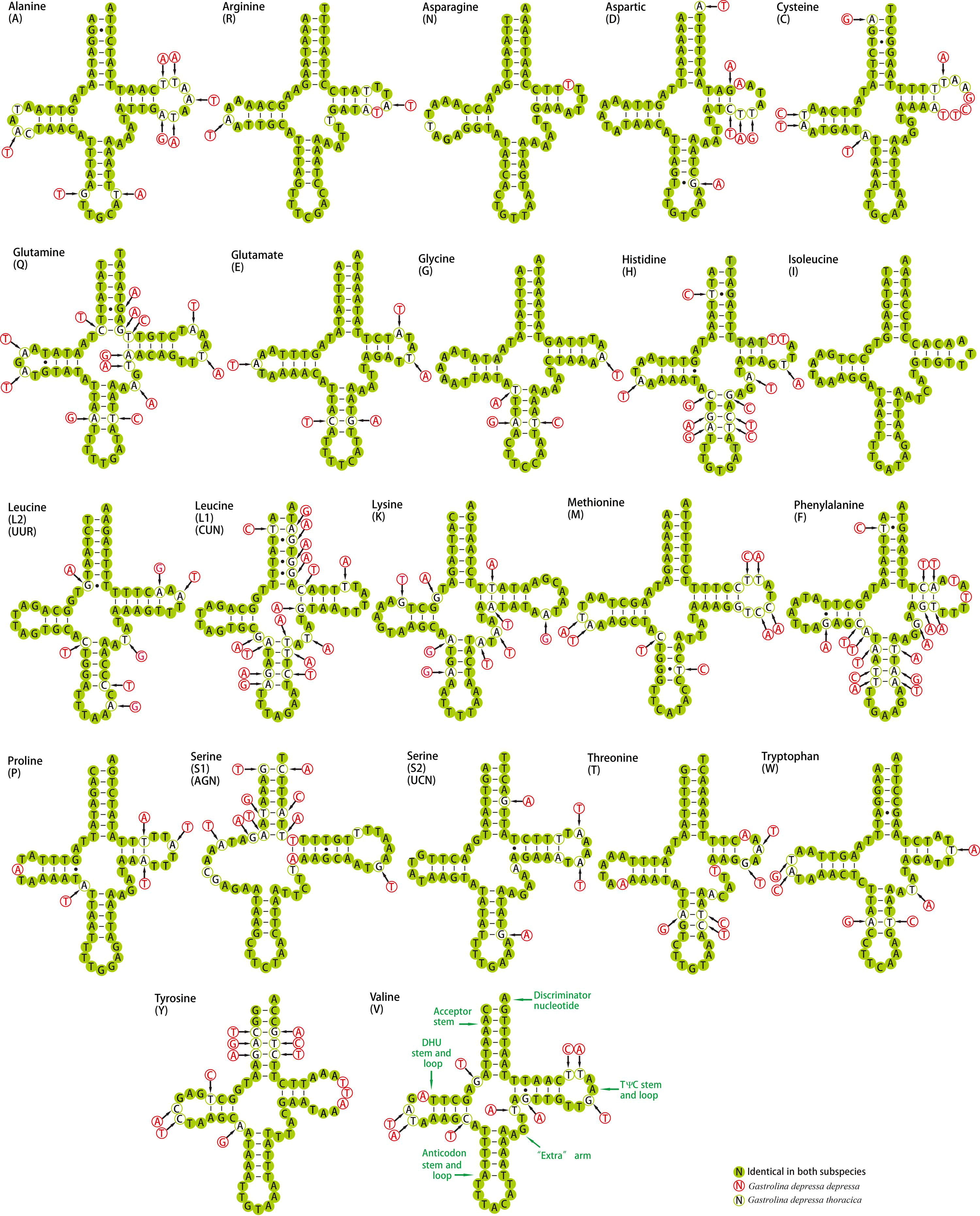

Supplement: Figure S2 — The nucleotide substitution pattern of each tRNA was modeled using as reference the structure determined for G. depressa thoracica. [file peerj-06-4919-s002.png]
