# Supplementary material for: The mitochondrial genomes of two walnut pests, Gastrolina depressa depressa and G. depressa thoracica (Coleoptera: Chrysomelidae), and phylogenetic analyses
Source: PeerJ. 2018 Jun 5;6:e4919. doi: 10.7717/peerj.4919 (PMC5993032; doi:10.7717/peerj.4919)
Supplement: Table S1 [file peerj-06-4919-s003.docx]

| **suborder** | **superfamily** | **Family** | **Subfamily** | **Species** | **ACC. Number** | **References** |
| --- | --- | --- | --- | --- | --- | --- |
| Polyphaga | Chrysomeloidea | Chrysomelidae | Bruchinae | *Acanthoscelides obtectus* | KX825864 | *Jie et al*., *2017* |
|  |  |  | Cassidinae | *Cassida viridis* | KX943468 | *Gómez*‐*Rodríguez et al*., *2015* |
|  |  |  |  | *Arescus labiatus* | JX220988 | *Timmermans et al*., *2015* |
|  |  |  |  | *Laccoptera ruginosa* | JX412753 | *Timmermans et al*., *2015* |
|  |  |  | Chrysomelinae | *Gastrolina depressa depressa* | MF198407 | This study |
|  |  |  |  | *Gastrolina depressa thoracica* | MF198406 | This study |
|  |  |  |  | *Gonioctena intermedia* | KX922881 | *Dierckxsens et al*., *2017* |
|  |  |  | Criocerinae | *Crioceris duodecimpunctata* | AF467886 | *Stewart* *& Beckenbach*, *2003* |
|  |  |  | Cryptocephalinae | *Cryptocephalus ramburii* | KX943509 | *Gómez*‐*Rodríguez et al*., *2015* |
|  |  |  |  | *Cryptocephalus lividimanus* | KX943495 | *Gómez*‐*Rodríguez et al*., *2015* |
|  |  |  |  | *Stylosomus rugithorax* | KX943428 | *Gómez*‐*Rodríguez et al*., *2015* |
|  |  |  |  | *Pachybrachis* sp. ReAss_5 | KX943419 | *Gómez*‐*Rodríguez et al*., *2015* |
|  |  |  | Donaciinae | *Plateumaris sericea* | KX943505 | *Gómez*‐*Rodríguez et al*., *2015* |
|  |  |  | Eumolpinae | *Pseudocolaspis* sp. PSE01 | JX412756 | *Timmermans et al*., *2015* |
|  |  |  | Galerucinae | *Diabrotica barberi* | KF669870 | *Coates*, *2014* |
|  |  |  |  | *Dibrotica virgifera virgifera* | KF658070 | *Coates*, *2014* |
|  |  |  |  | *Galeruca daurica* | KR025478 | *Zhou et al*., *2016* |
|  |  |  |  | *Paleosepharia posticata* | KY195975 | *Wang & Tang*, *2017* |
|  |  |  | Spilopyrinae | *Spilopyra sumptuosa* | JX220997 | *Timmermans et al*., *2015* |
|  |  | Cerambycidae | Cerambycinae | *Massicus raddei* | KC751569 | *Wang et al*., *2014* |
|  |  |  |  | *Obrium* sp. NS-2015 | KT945156 | *Song et al*., *2015* |
|  |  |  |  | *Xylotrechus grayii* | KM112084 | Direct Submission |
|  |  |  | Lamiinae | *Anoplophora glabripennis* | DQ768215 | *An et al*., *2004* |
|  |  |  |  | *Anoplophora chinensis* | KT726932 | *Li et al*., *2015* |
|  |  |  |  | *Apriona swainsoni* | KX184801 | Direct Submission |
|  |  |  |  | *Batocera lineolata* | JN986793 | Direct Submission |
|  |  |  |  | *Thyestilla gebleri* | KY292221 | Direct Submission |
|  |  |  | Lepturinae | *Leptura arcuata* | KY796051 | Unpublished |
|  |  |  |  | *Stictoleptura succedanea* | KY796052 | Unpublished |
|  |  |  | Necydalinae | *Necydalis ulmi* | JX220989 | *Timmermans et al*., *2015* |
|  |  |  | Prioninae | *Prioninae* sp. MJTNT-2012 | JX220991 | *Timmermans et al*., *2015* |
|  |  | Disteniidae | Disteniinae | *Disteniazteca fimbriata* | JX221000 | *Timmermans et al*., *2015* |
|  |  | Orsodacnidae | Orsodacninae | *Orsodacne lineola* | JX220994 | *Timmermans et al*., *2015* |
|  |  | Vesperidae | Philinae | *Spiniphilus spinicornis* | KT781589 | *Nie et al*., *2015* |
| Archostemata |  | Ommatidae |  | *Tetraphalerus bruchi* | EU877953 | *Sheffield et al*., *2008* |
| Adephaga | Caraboidea | Carabidae |  | *Abax parallelepipedus* | KT876877 | *Linard et al*., *2016* |

**References**

**An YL, Huang XM, Yang XJ, Lin XJ, Ji BZ, Wang DB, Victor CM. 2004.** A study on mtDNA sequence and its genetic characteristics of *Anoplophora glabripennis* and its sibling species. *Journal of Nanjing Forestry University* **28(4)**:6–12.

**Coates BS. 2014.** Assembly and annotation of full mitochondrial genomes for the corn rootworm species, *Diabrotica virgifera virgifera* and *Diabrotica barberi* (Insecta: Coleoptera: Chrysomelidae), using Next Generation Sequence data. *Gene* 542(2):190–197.

**Dierckxsens N, Mardulyn P, Smits G. 2017.** NOVOPlasty: de novo assembly of organelle genomes from whole genome data. *Nucleic Acids Research* **45(4)**:e18.

**Gómez-Rodríguez C, Crampton-Platt A, Timmermans MJTN, Baselga A, Vogler AP. 2015.** Validating the power of mitochondrial metagenomics for community ecology and phylogenetics of complex assemblages. *Methods in Ecology and Evolution* **6(8)**:883–894.

**Jie Y, Hong Y, Dai RH. 2017.** Characterization of the complete mitochondrial genome of *Acanthoscelides Obtectus* (Coleoptera: Chrysomelidae: Bruchinae) with phylogenetic analysis. *Genetica* **145(4–5)**:397–408.

**Li WB, Yang XJ, Qian L, An YL, Fang J. 2015.** The complete mitochondrial genome of the citrus long-horned beetle, Anoplophora chinensis (Coleoptera: Cerambycidae). *Mitochondrial DNA*. *Part A*, *DNA Mapping*, *Sequencing*, *and Analysis* **27(6)**:4665–4667.

**Linard B, Arribas P, Andújar C, Crampton-Platt A, Vogler AP. 2016.** Lessons from genome skimming of arthropod-preserving ethanol. *Molecular Ecology Resources* **16(6)**:1365–1377.

**Nie RE, Lin MY, Xue HJ, Bai M, Yang XK. 2015.** Complete mitochondrial genome of *Spiniphilus spinicornis* (Coleoptera: Vesperidae: Philinae) and phylogenetic analysis among Cerambycoidea. *Mitochondrial DNA*. *Part A*, *DNA Mapping*, *Sequencing*, *and Analysis* **28(1)**:145–146.

**Sheffield NC, Song H, Cameron SL, Whiting MF. 2008.** A comparative analysis of mitochondrial genomes in Coleoptera (Arthropoda: Insecta) and genome descriptions of six new beetles. *Molecular Biology and Evolution* **25(11)**:2499–2509.

**Song N, Zhang H, Yin XM, Lin AL, Zhai Q. 2015.** The complete mitochondrial genome sequence from the longicorn beetle *Obrium* sp. (Coleoptera: Cerambycidae). *Mitochondrial DNA*. *Part A*, *DNA Mapping*, *Sequencing*, *and Analysis* **28(3)**:326–327.

**Stewart JB, Beckenbach AT. 2003.** Phylogenetic and genomic analysis of the complete mitochondrial DNA sequence of the spotted asparagus beetle *Crioceris duodecimpunctata*. *Molecular Phylogenetics and Evolution* **26(3)**:513–526.

**Timmermans MJ, Barton C, Haran J, Ahrens D, Culverwell CL, Ollikainen A, Dodsworth S, Foster PG, Bocak L, Vogler AP. 2015.** Family-level sampling of mitochondrial genomes in Coleoptera: compositional heterogeneity and phylogenetics. *Genome Biology and Evolution* **8(1)**:161–175.

**Wang QQ, Tang GH. 2017.** Genomic and phylogenetic analysis of the complete mitochondrial DNA sequence of walnut leaf pest Paleosepharia posticata (Coleoptera: Chrysomeloidea). *Journal of Asia*-*Pacific Entomology* **20(3)**:840–853.

**Wang YT, Liu YX, Tong XL, Ren QP, Jiang GF. 2014.** The complete mitochondrial genome of the longhorn beetle, *Massicus raddei*. *Mitochondrial DNA*. *Part A*, *DNA Mapping*, *Sequencing*, *and Analysis* **27(1)**:209–211.

**Zhou XR, Han HB, Pang BP, Zhang PF. 2016.** The complete mitochondrial genome of *Galeruca daurica* (Joannis) (Coleoptera: Chrysomelidae). *Mitochondrial DNA*. *Part A*, *DNA Mapping*, *Sequencing*, *and Analysis* **27(4)**:2891–2892.
