# Supplementary material for: The mitochondrial genomes of two walnut pests, Gastrolina depressa depressa and G. depressa thoracica (Coleoptera: Chrysomelidae), and phylogenetic analyses
Source: PeerJ. 2018 Jun 5;6:e4919. doi: 10.7717/peerj.4919 (PMC5993032; doi:10.7717/peerj.4919)
Supplement: Table S2 [file peerj-06-4919-s004.docx]

| **Model** | **Parameter** | **Values** |
| --- | --- | --- |
| TVM+I+G | partition | 012314 |
|  | feqA | 0.3724 |
|  | feqC | 0.0927 |
|  | feqG | 0.0794 |
|  | feqT | 0.4556 |
|  | R(a) | 0.7570 |
|  | R(b) | 6.1568 |
|  | R(c) | 0.9416 |
|  | R(d) | 4.2990 |
|  | R(e) | 6.1568 |
|  | R(f) | 1.0000 |
|  | p-inv | 0.2400 |
|  | gamma | 0.5890 |
| MtArt+I+G+F | Alpha | 0.489 |
|  | Alpha-Inv | 0.728 |
|  | Inv-Alpha | 0.204 |
|  | Inv | NaN |
