# Supplementary material for: The mitochondrial genomes of two walnut pests, Gastrolina depressa depressa and G. depressa thoracica (Coleoptera: Chrysomelidae), and phylogenetic analyses
Source: PeerJ. 2018 Jun 5;6:e4919. doi: 10.7717/peerj.4919 (PMC5993032; doi:10.7717/peerj.4919)
Supplement: Table S3 — Genes encoded by H-Strand were in bold. [file peerj-06-4919-s005.docx]

| **Gene** | ***G*. *depressa thorcica*** | ***G*. *depressa depressa*** | ***Paleosepharia posticata*** | ***Galeruca daurica*** | ***Agasicles hygrophila*** |
| --- | --- | --- | --- | --- | --- |
| ***nad2*** | 1 | 2 | 1 | 1 | 1 |
| ***cox1*** | 0 | 0 | 0 | 0 | 0 |
| ***cox2*** | 2 | 2 | 2 | 2 | 2 |
| ***atp8*** | 0 | 0 | 0 | 0 | 0 |
| ***atp6*** | 1 | 1 | 1 | 1 | 1 |
| ***cox3*** | 1 | 1 | 1 | 1 | 1 |
| ***nad3*** | 1 | 1 | 1 | 1 | 1 |
| ***nad6*** | 3 | 1 | 1 | 1 | 1 |
| ***cob*** | 4 | 4 | 4 | 4 | 4 |
| *nad1* | 9 | 5 | 6 | 5 | 5 |
| *nad5* | 6 | 6 | 8 | 9 | 11 |
| *nad4* | 6 | 8 | 5 | 9 | 8 |
| *nad4l* | 1 | 2 | 1 | 1 | 1 |
