# Supplementary material for: The mitochondrial genomes of two walnut pests, Gastrolina depressa depressa and G. depressa thoracica (Coleoptera: Chrysomelidae), and phylogenetic analyses
Source: PeerJ. 2018 Jun 5;6:e4919. doi: 10.7717/peerj.4919 (PMC5993032; doi:10.7717/peerj.4919)
Supplement: Table S4 [file peerj-06-4919-s006.docx]

| **tRNA** | **amino acid** | **total length** | **identical positions** | **%INUC** |
| --- | --- | --- | --- | --- |
| *trnA* | Alanine | 67 | 56 | 83.58 |
| *trnC* | Cysteine | 64 | 53 | 82.81 |
| *trnD* | Aspartate | 64 | 58 | 90.63 |
| *trnE* | Glutamate | 63 | 58 | 92.06 |
| *trnF* | Phenylalanine | 66 | 50 | 75.76 |
| *trnG* | Glycine | 64 | 60 | 93.75 |
| *trnH* | Histidine | 64 | 52 | 81.25 |
| *trnI* | Isoleucine | 65 | - | - |
| *trnK* | Lysine | 71 | 62 | 87.32 |
| *trnL1* | Leucine(CUN) | 65 | 49 | 75.38 |
| *trnL2* | Leucine(UUR) | 65 | 58 | 89.23 |
| *trnM* | Methionine | 70 | 59 | 84.29 |
| *trnN* | Asparagine | 64 | 63 | 98.44 |
| *trnP* | Proline | 64 | 59 | 92.19 |
| *trnQ* | Glutamine | 69 | 56 | 81.16 |
| *trnR* | Arginine | 63 | 61 | 96.83 |
| *trnS1* | Serine(AGN) | 67 | 54 | 80.60 |
| *trnS2* | Serine(UCN) | 67 | 61 | 91.04 |
| *trnT* | Threonine | 64 | 56 | 87.50 |
| *trnV* | Valine | 69 | 59 | 85.51 |
| *trnW* | Tryptophan | 64 | 58 | 90.63 |
| *trnY* | Tyrosine | 67 | 54 | 80.60 |
